# Thyrotomy for Cancer of the Larynx, with Report of Eleven Cases

**Published:** 1913-10

**Authors:** D. Crosby Greene

**Affiliations:** Boston


### Thyrotomy for Cancer of the Larynx, With Report of Eleven Cases.

DR. D. CROSBY GREENE. Boston: In a paper presented to this association in 1906 we reported the results of an investigation of the lymphatic drainage of the larynx by means of submucous injections of methylen blue and mercury. The results obtained are confirmatory of those reported by others in showing that the network of lymphatic vessels which extends beneath the mucous membrane throughout the interior of the larynx is richer in the number and size of the vessels in the supraglottic region, relatively poorer in the subglottic portion, while on the vocal cords the vessels are very small and widely separated. These anatomic facts account for the slow growth and late development of the disease in the cervical lymph nodes in cases of epithelioma of the cords, and furnish an argument for the possibility of cure in early cases by the operation of thyrotomy and excision of the growth with a wide margin of healthy tissue. This is supported by the result of the operation in the hands of numerous operators, both in this country and abroad, so that at the present time it is almost universally recognized as the proper procedure for the treatment of early intrinsic cancer of the larynx. Certain details of the technic have an important bearing on the immediate and after-results of the operation. The steps of the operation are: 1. Ether by inhalation, preceded an hour before by ¼ grain of morphine and 1/150 grain of atropin. 2. With the head slightly extended a median incision is made, extending from the lower border of the hyoid bone to the lower border of the cricoid cartilage. This incision is carried down through the prethyroid muscles until the thyroid and cricoid cartilages and cricothyroid membrane have been definitely exposed. 3. A one per cent solution of cocain is injected through the cricothyroid membrane into the cavity of the larynx. 4. The patient is now placed in the Trendelenberg position and a thick pad placed under the shoulders to bring the larynx into prominence. 5. The cricothyroid membrane is next incised in the median line, and through this incision a swab of ten per cent solution of cocain is introduced and applied to the laryngeal mucous membrane. 6. The thyroid cartilage, after a pause of five minutes, is divided from below upwards. In young subjects this may be done with a knife, but in the majority of cases where the cartilage has become ossified, it is best to use strong curved scissors with dull points. 7. The thyroid wings are now widely retracted and an examination of the growth made under good illumination. 8. Beginning at the free margin of the thyroid cartilage, on the affected side in front of the growth, the internal perichondrium is elevated from off the cartilage with a sharp elevator from before backwards to a line well behind the limits of the growth as well as above and below it. All the soft structures are thus freed from the underlying cartilage. 9. Parallel horizontal incisions are now made with scissors above and below the growth. These incisions are carried about one-half inch back of the posterior limit of the growth. 10. The growth with its surrounding tissue is now entirely removed with a wire snare by which the posterior attachments are severed. Much depends on the proper selection of cases. When the growth is so extensive, even though confined within the cavity of the larynx, that the larynx cannot be opened without cutting into the growth, recurrence is not only possible but probable.—*Abstract of papers read at the Thirty-fifth Annual Congress of the American Laryngological Association, at Washington, D. C., May 5-7, 1913. Reported by Emil Meyer, abstract editor.*

